# Hospital Centralisation at Aberdeen

**Published:** 1887-01-08

**Authors:** 


### HOSPITAL CENTRALISATION AT ABERDEEN.

The president and managers of the Aberdeen Infirmary are about to make application to Parliament for an act to repeal certain charters, and to provide for the incorporation of the present managers in a new form. Powers are also sought for the inclusion, in this same corporation, of all hospitals, asylums, or dispensaries established or hereafter to be established, and in particular, the Hospital for the Relief of Incurables, the Ophthalmic Institution, the Dispensary, and the Hospital for Sick Children. This is a large measure as well as a new departure, and demands the most careful consideration on the part of all who are interested in the management of hospitals. Primarily, it affects the people of Aberdeen, and they may be trusted to manage their own affairs with that prudence which is not the least conspicuous of Scottish virtues. It is not, however, a matter of small importance to others even in England, because a precedent, once established, exercises a certain authority in similar cases, as well in municipal and other business as in legal affairs. It is satisfactory to note that the directors of the Dispensary have for the time declared against the scheme, because their opposition will ensure a full and adequate discussion of the subject before the final decision is arrived at, and such discussion is eminently desirable from every point of view. The significance of the scheme is this, that it proposes to place all the public charities of Aberdeen of every kind under *one central authority*, and to include with these the lunatic asylums, which are not charitable institutions.

The object of the proposed Act, we suppose, is to secure two things, both of which are dear to the Scottish mind, viz., *Efficiency* and *Economy.* These are the stand-points from which to view the proposal, and from which to consider it in its application both to Aberdeen and to other places. Of these, efficiency is first in the order of importance. An institution *must be efficient;* it should also, if possible, be *economical.* We have to consider, therefore, whether the placing of so many and such various institutions as hospitals, dispensaries, ophthalmic hospitals, lunatic asylums, and convalescent institutions under one central authority will conduce to their efficiency. And the first thing that strikes one, in instituting such a consideration, is that the central authority will not lack work. No committee sitting once a month, or even once a week, can properly conduct the affairs of so many and such varied institutions. The members must either devote their whole time to their duties, or they must delegate them in detail to others, they themselves sitting as a final authority or court of appeal. We do not know whether it is proposed to make the central authority a paid or an honorary body; and this is a point of no little importance. If, as we presume, it is to be honorary, then a great part of its duties must be performed by delegation; and it becomes a question whether a number of bodies, subordinate to one central authority, or several independent organisations, responsible only to the public, will best perform the duties to be done. As regard *efficiency*, therefore, it is by no means clear that the proposed change will be advantageous: there is, at any rate, much to be said on both sides.

The next question is that of *economy*; and this, though second in importance to that of *efficiency*, is yet in itself worthy of the most thoughtful consideration. In these times, the strain upon the resources of the public is so great and so continuous, that he who teaches us how to make one penny do the duty of two, is as much deserving of honour as he who makes two blades of grass to grow where only one grew before. In other words, diminished expenditure without lessened efficiency conduces to wealth as much as increased production. We, at any rate, are not likely to undervalue economy in the administration of charities. Will the Aberdeen scheme—the concentration of the powers of all the charities in one corporation—tend to promote economy? And if so, to what extent? There seems reason to believe that an affirmative answer may be given to this question. Where there are a variety of institutions, all performing very similar and in some respects identical functions, there must be more or less overlapping among them. Not only so, but there is a tendency to increase the number of the institutions themselves: and, for each one in its laudable desire to obtain its full share of public recognition, to develope itself beyond the actual necessities of the case. The frog that wanted to become an ox, is not without descendants and imitators even among the public charities. All men and managers are mortal, and there is an universal and unconquerable objection in every mind to "playing second fiddle."

But sound policy demands that in charity, as in other things, there shall not be two institutions where one would serve the purpose; and that two or more hospitals shall not be performing functions in duplicate, and even in triplicate. To take a single instance—Why, in such a city as Aberdeen, should there be a separate special hospital for diseases of the eye, when those diseases can be just as efficiently treated by creating a special department at the General Hospital? By the creation of special departments at general hospitals, a whole class of special institutions are rendered superfluous, and an enormous saving of resources is effected without any diminution of efficiency. From the point of view of economy, therefore, the Aberdeen scheme may be regarded with decided favour.

There is one more point to be taken into review. All this is proposed to be done by "Act of Parliament." Perhaps in the case of Aberdeen, where Parliament has, at least twice before, been called upon to exercise its authority in the affairs of the Infirmary, a third appeal to that ridden-to-death hack may be absolutely unavoidable. But, speaking generally, it seems to us neither necessary nor desirable that charitable affairs should be carried to the House of Commons. If the constituents of the various charities of Aberdeen are for any reason unable or unwilling to manage the affairs of the charities, an appeal to the Town Council, or to a specially appointed Committee of Judicious Persons might be had resort to. In general, Parliament has neither the time nor the disposition nor the knowledge, to deal competently with such purely local questions, and for this reason the Limited Liability Acts now provide for the incorporation of charities without resort to special Acts and Royal Charters. In most cases, it seems best that those persons who regularly furnish the resources for charitable objects should, in the last resort, determine the method and scope of their administration.

